# TAp63 regulates oncogenic miR-155 to mediate migration and tumour growth

**DOI:** 10.18632/oncotarget.1228

**Published:** 2013-08-31

**Authors:** Sam Mattiske, Kristen Ho, Jacqueline E. Noll, Paul M. Neilsen, David F. Callen, Rachel J. Suetani

**Affiliations:** ^1^ Cancer Therapeutics Laboratory, Centre for Personalised Cancer Medicine, University of Adelaide, Australia

**Keywords:** miR-155, TAp63, ΔNp63, cancer, tumorigenesis, migration

## Abstract

miR-155 is an oncogenic microRNA which is upregulated in many solid cancers. The targets of miR-155 are well established, with over 100 confirmed mRNA targets. However, the regulation of miR-155 and the basis of its upregulation in cancer is not well understood. We have previously shown that miR-155 is regulated by p63, and here we investigate the role of the major p63 isoforms TAp63 and ΔNp63 in this regulation. When the TAp63 isoform was knocked down, or exogenously overexpressed, miR-155 levels were elevated in response to TAp63 knockdown or reduced in response to TAp63 overexpression. The ΔNp63 isoform is shown to directly bind to the p63 response element on the miR-155 host gene, and this binding is enriched when TAp63 is knocked down. This could indicate that TAp63 prevents ΔNp63 from binding to the miR-155 host gene. The knockdown of TAp63, and the subsequent elevation of miR-155, enhances migration and tumour growth similar to that seen when directly overexpressing miR-155. The migratory phenotype is abrogated when miR-155 is inhibited, indicating that miR-155 is responsible for the phenotypic effect of TAp63 knockdown.

## INTRODUCTION

MicroRNAs (miRs) are short noncoding RNAs that fine-tune gene regulation by inhibiting translation of their target mRNAs [1]. A single miR can regulate many target genes through specific binding to short seed sequences generally located in a gene's 3' untranslated region (UTR).

Due to their ability to regulate many genes in multiple different pathways, aberrant expression of a miR can have severe consequences. miR-155 is an oncogenic miR upregulated in multiple cancers including leukaemia, lymphoma, colon, thyroid, pancreatic, gastric, lung and breast cancer [2-5]. Elevated miR-155 expression is associated with poor prognosis in pancreatic and lung cancers [6, 7], while in breast cancers miR-155 expression contributes to epithelial cell plasticity as well as driving invasion [8, 9]. Other miRs can have an opposite, tumour suppressive, role such as miR-218 inhibiting cell migration and invasion [10].

The downstream targets of miR-155 are well established with over one hundred validated [11]. These mRNA targets are involved in a number of diverse pathways. For example, miR-155 has been shown to regulate the angiogenesis promoter *HIF-1α* [12], the apoptosis factors *FADD* and *CASP3* [13], and members of the *SMAD* family of genes which are involved in epithelial to mesenchymal transition (EMT) [14-16].

However, the mechanisms by which miR-155 is upregulated in cancer is not understood, as the pathways regulating miR-155 expression have not been fully explored. miR-155 is encoded by the *BIC* (also known as *MIR155*) gene [17], a spliced and adenylated but non-protein coding gene that was originally implicated in lymphoma [18]. There is data relating to the regulation of miR-155 in the immunological setting where miR-155 is regulated by cytokines, interleukins and interferon such as ERK/JNK [14] and STAT1 [19]. The basis for the upregulation of miR-155 in cancer is unknown. In breast cancer it has been shown that the TGF-β epithelial-mesenchymal transition pathway upregulates miR-155 through SMAD4 [8]. Additionally, BRCA1 has been shown to epigenetically repress the expression of miR-155[20], and this repression can be lost in breast cancer [21]. Epigenetic repression of another miR, miR-211, has been shown to control apoptosis, chemosensitivity and radiosensitivity in glioma [22], therefore the regulation of miR-155 may be similarly significant. Recently, in colon cancer miR-155 expression was shown to be promoted by S100P/RAGE through Activator Protein-1 (AP-1) stimulation [23]. In other solid cancers the exact mechanisms of miR-155 upregulation have not been reported.

Previously, we showed that miR-155 can be repressed by direct binding of either p53 or p63 to response elements in the host gene, *BIC* [9]. p63 is a member of the p53 family of transcriptional regulators and shares sequence homology with the transactivating, DNA binding, and oligomerisation domains of p53. While this may suggest a similar role to p53, the “guardian of the genome”, it has emerged that p63 is a regulator of epithelial development [24, 25]. There are two major isoforms of p63, resulting from alternative transcriptional start sites. The full-length TAp63 isoform contains an N-terminal transactivation domain whereas ΔNp63 is a truncated version that lacks this domain [26]. Curiously, the two isoforms exert opposing functions in cancer with TAp63, similarly to p53, functioning as a tumour suppressor and inducing cell death and cell cycle arrest [27]. Knockout of TAp63 in mice, results in the spontaneous formation of metastatic tumours [28]. In contrast, ΔNp63 is oncogenic and often overexpressed in cancer [29].

It is not known which of the p63 isoforms mediates the regulation of miR-155 expression. This study investigates the role of p63 isoforms in the regulation of miR-155 expression and the resulting phenotypic consequences.

## RESULTS

### The expression of miR-155 is regulated by TAp63

**Figure 1:**
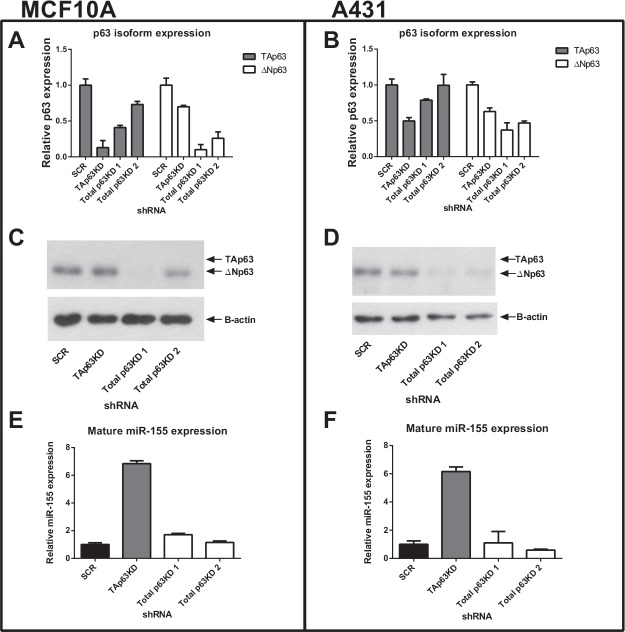
Knockdown of TAp63 induces miR-155 expression MCF10A breast epithelial cells and A431 skin carcinoma cells were transduced with shRNAs targeting TAp63, total p63 (2 independent shRNAs) or a non-silencing control shRNA (SCR). A, B: p63 isoform RNA expression levels of MCF10A and A431 knockdown cell lines were measured using isoform-specific qRT-PCR. C, D: Western blot analysis of p63 protein in MCF10A and A431 cells treated with p63 shRNA hairpins. The anti-p63 antibody (H-129) detects both the TAp63 and ΔNp63 isoforms although both MCF10A and A431 cells contain ~99% ΔNp63 isoform. E, F: Mature miR-155 expression levels of MCF10A and A431 cells treated with p63 shRNA hairpins were measured using specific Taqman miRNA probes.

In order to investigate the regulation of miR-155 expression by the p63 isoforms, isoform specific knockdowns were created in two cell lines using a shRNA approach. The MCF10A breast non-malignant epithelial cell line and A431 skin carcinoma cell line were selected due to their high endogenous p63 (predominantly ΔNp63 isoform) and low miR-155 expression levels (Supplementary Figure 1). Three shRNA hairpins targeting the p63 transcript and a non-silencing control shRNA were used to knock down p63 expression in these cell lines, with one p63 shRNA selectively targeting the TAp63 isoform and two independent shRNAs targeting both isoforms. Unfortunately we were not able to create a ΔNp63 specific knockdown. Knockdown was measured by isoform-specific real-time qPCR (Figure 1A, B) and by western blot (Figure 1C, D). TAp63 protein is expressed at a level undetectable by western blot in these cells but qPCR showed the specific shRNA hairpin reduced expression of the TAp63 isoform in MCF10A cells by 85% and in A431 cells by 50%.

The expression level of mature miR-155 was measured by real-time qPCR using specific Taqman probes. In MCF10A cells, miR-155 expression levels increased by over six-fold when TAp63 was knocked down (Figure 1E). Similar results were observed in A431 cells where the TAp63 knockdown resulted in a six-fold increase in miR-155 expression (Figure 1F). The lack of influence of knockdown of the ΔNp63 isoform (up to 90% in MCF10A and 60% in A431) on MIR155 expression implies a specific repressive role of the full-length isoform of p63 on MIR155 expression.

### Exogenous expression of TAp63 inhibits miR-155 expression

**Figure 2:**
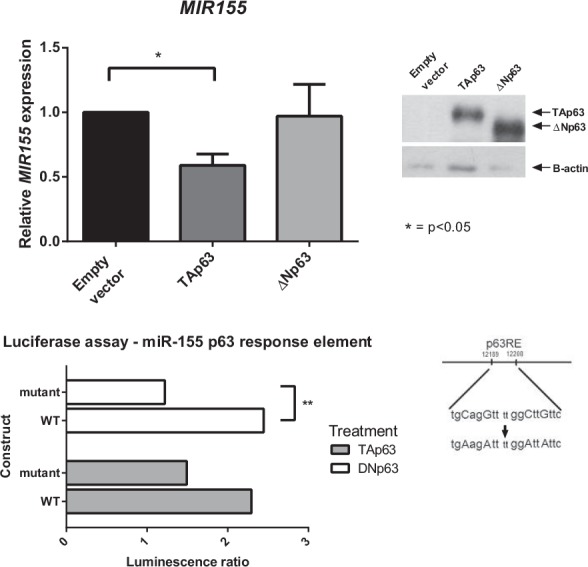
Exogenous expression of TAp63 inhibits miR-155 expression TAp63, ΔNp63 or empty vector were transiently transfected into BT549 breast tumour cells. Expression levels of *MIR155* mRNA levels were measured by real-time qPCR. p63 isoform expression in these cells was validated using western blot.

To further investigate the role of p63 isoforms on MIR155 expression, the two p63 isoforms were each over-expressed in the BT549 breast tumour cell line, which endogenously expresses a high level of miR-155 (Supplementary Figure 1) and low endogenous p63. miR-155 expression levels, analysed by real-time qPCR (Figure 2), were significantly decreased by over-expression of TAp63, while over-expression of ΔNp63 did not significantly change miR-155 expression levels. Therefore both knockdown and over-expression of TAp63 isoforms are consistent with the TAp63 isoform having a repressive regulatory effect on miR-155 expression levels.

### The ΔNp63 isoform directly binds the miR-155 p63RE and drives expression

**Figure 3:**
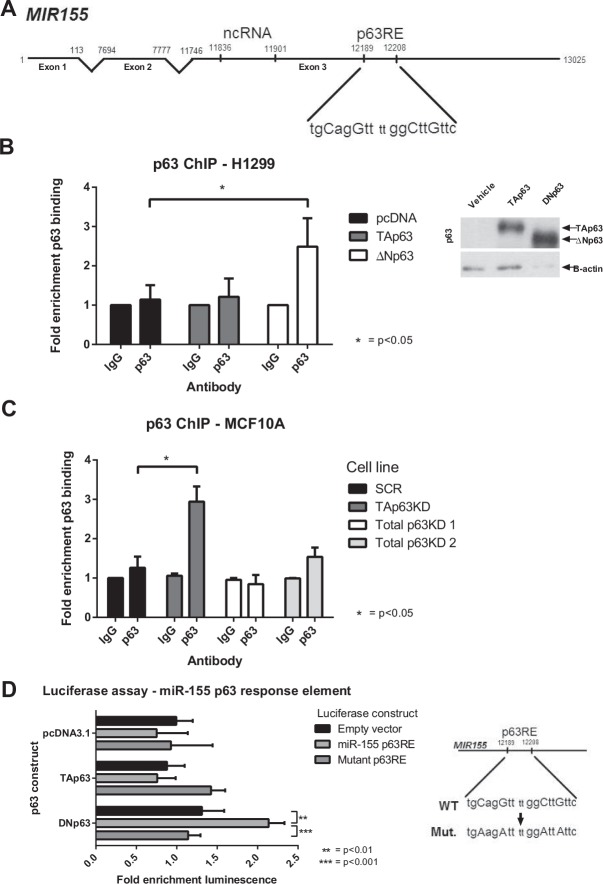
ΔNp63 binds to the miR-155 host gene and drives expression A: The p63 response element and miR-155 ncRNA are located in exon 3 of the miR-155 host gene. B, C: ChIP assays were performed in H1299 cells transfected with TAp63, ΔNp63 or empty vector overexpression constructs (B) and in MCF10A cells with p63 isoforms knocked down by shRNA (C). DNA–p63 complexes were immunoprecipitated from samples using a p63 antibody (or IgG control). Fold enrichment of binding to the p63-RE within the MIR155HG gene was determined as compared to IgG controls. Protein expression of p63 isoforms in the H1299 cell lysate was determined using western blot. D: Dual luciferase reporter assay of empty pGL3 Basic vector, pGL3-p63RE or pGL3-mutantp63RE co-transfected with pRL-TK and either empty vector, TAp63 or ΔNp63.

Previously we showed that p53 and p63 bind to the response element present in exon 3 of the miR-155 host gene (*MIR155* or *BIC*, schematic diagram shown in Figure 3A)[9]. To determine which p63 isoform was binding at this site, p63 ChIP assays were completed on H1299 cells exogenously expressing either TAp63 or ΔNp63 (Figure 3B). Binding at the p63-RE was enriched when the ΔNp63 isoform was expressed in these cells, while in cells exogeneously expressing the TAp63 isoform there was no significant difference between TAp63 binding and vector control. To explore this further, p63 ChIP assays used MCF10A cells with either the TAp63 isoform, or total p63 expression, knocked down with shRNAs (Figure 3C). Binding of p63 to the *MIR155* p63 response element was enriched in the TAp63 specific knockdown, as compared to the IgG and SCR control. Together these two approaches are consistent with ΔNp63 binding to the response element on *MIR155*. This was unexpected considering the previous findings showing a specific repressive effect of only the TAp63 isoform on the expression of miR-155.

In order to confirm that ΔNp63 has an effect on the miR-155 p63 response element and is able to drive expression of miR-155, the ability of the p63 isoforms to drive expression of a luciferase reporter gene was assessed in 293T cells. The ΔNp63 isoform was able to drive luciferase activity through the miR-155 p63 response element, as compared to empty luciferase vector and mutant response element (Figure 3D). The luciferase activity of TAp63 was not significantly different between wildtype and mutant response element, indicating that it is the ΔNp63 isoform that is acting upon the miR-155 host gene p63 response element.

### Release of miR-155 from TAp63 regulation drives migration

To investigate the relative contribution of miR-155 to the biological role of p63 as a tumour suppressor, the expression of miR-155 and p63 were modulated in MCF10A and A431 cells.

**Figure 4:**
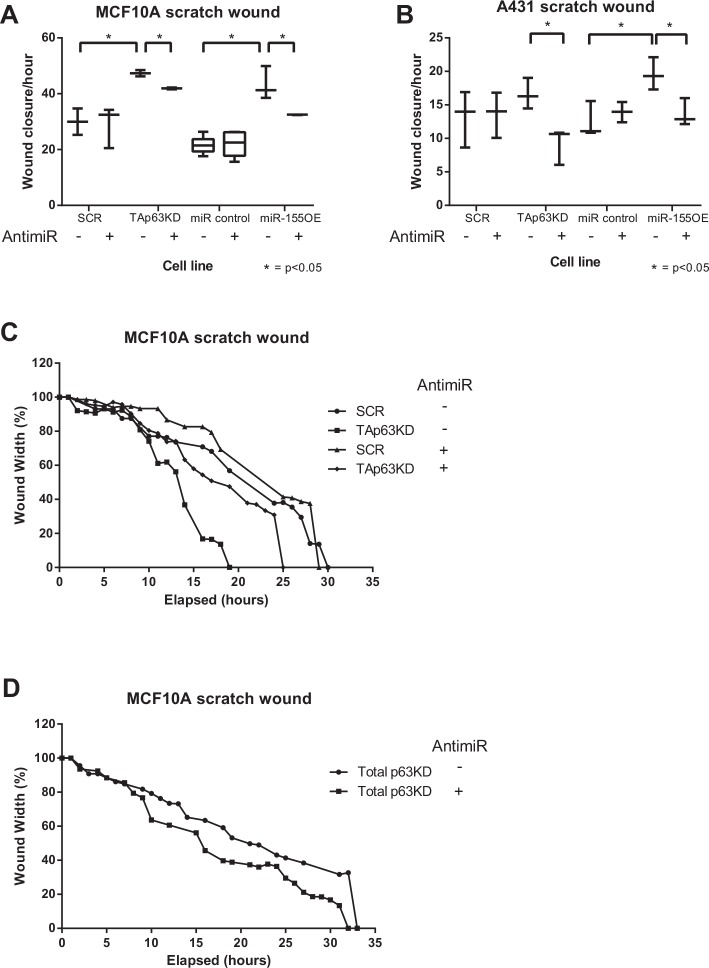
Release of miR-155 from TAp63 regulation drives migration A,B: The ability of MCF10A or A431 SCR, TAp63KD, miR control and miR-155 overexpressing cells to migrate with application of anti-miR-155 inhibitor (+) or a non-targeting anti-miR (−) was determined by a scratch-wound assay. Wound width was calculated using Incucyte software. C, D: Representative real time wound width data for SCR, TAp63KD (C) and total p63KD (D).

The migratory abilities of MCF10A and A431 cells with either TAp63 knockdown or miR-155 overexpression were determined using a scratch wound assay (Figure 4). The miR-155 levels following TAp63 knockdown were comparable to the levels seen with miR-155 overexpression cells (Supplementary Figure 2). TAp63 knockdown, or miR-155 over-expression, both increased the migratory potential of MCF10A and A431 cells (4A, C). In a “rescue treatment” of both cells lines use of anti-miR-155 inhibited this migratory phenotype, indicating that this phenotype is dependent on miR-155. Total p63 knockdown had no effect on migration (4D, Supplementary Figure 3). These data suggest that the increase in migratory potential resulting from TAp63 knockdown is largely caused by the resulting increase in miR-155 expression.

### TAp63 knockdown and miR-155 overexpression enhance tumour growth

**Figure 5:**
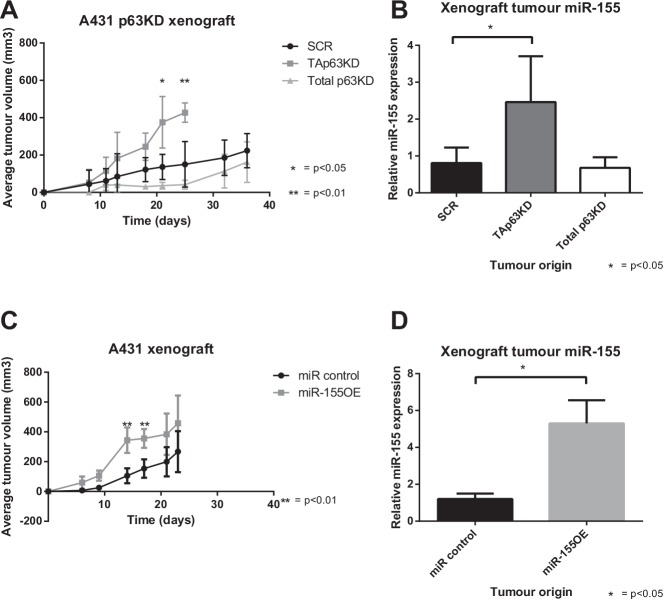
TAp63 knockdown and miR-155 overexpression enhance tumour growth A: A431 cells bearing TAp63 knockdown, total p63 knockdown or scrambled control were xenografted into nude mice, and the tumour volume measured for up to 36 days. B: Mature miR-155 levels of snap frozen xenograft tumours, analysed by miR-155 specific probe and compared to miR-16. C: A431 cells bearing miR-155 overexpression or a miR control were xenografted into nude mice, and the tumour volume measured for up to 23 days. D: Mature miR-155 levels of snap frozen xenograft tumours, analysed by miR-155 specific probe and compared to miR-16.

The effect of TAp63, total p63 knockdown, and miR-155 overexpression on tumour growth was analysed in a xenograft model where modified A431 cells were injected subcutaneously into Balb/C nude mice, and the tumour volume monitored over 3 to 5 weeks. A431 cells (and not MCF10A cells) were selected due to their previous established tumourigenicity in xenograft studies [30]. TAp63 knockdown enhanced tumour growth, compared with both control and total p63 knockdown (Figure 5A), where after 3 weeks the average tumour volume of mice bearing TAp63 knockdown was approximately twice that of the total p63 knockdown and control groups. The overexpression of miR-155 also enhanced tumour growth, but there was greater variation within the miR-155 overexpression and control groups (Figure 5C). This suggests that the effect of TAp63 knockdown on tumour growth is at least partly attributable to the resulting increase in miR-155 expression.

## DISCUSSION

It has been observed that miR-155 is up-regulated in numerous solid cancers [2-5] but the mechanism by which this occurs is not well understood. Previously we showed that *MIR155* is under the regulation of p63 [9]. Here we show for the first time the effect of individual p63 isoforms on miR-155 expression.

p63 isoforms, and the ratios between their expression, have been shown to have a role in cancer [31]. The full-length TAp63 isoform is a tumour suppressor [27], so it would be expected to suppress miR-155 expression. Conversely, ΔNp63 has oncogenic effects [29] and would be expected to drive or enhance miR-155 expression. In two separate epithelial cell lines, knockdown of the TAp63 isoform greatly induced miR-155 expression, while total p63 knockdown had little or no effect (Figure 1). This indicates that miR-155 is under the regulation of TAp63 in some way, either directly or indirectly. In light of miR regulation TAp63 is known to transactivate DICER and thus miRs in general [28]. The findings presented here are not a result of aberrant DICER expression, as when TAp63 is knocked down (hence reducing DICER transactivation by TAp63) global miR biogenesis would be expected to be reduced along with DICER protein levels. Contrary to this, miR-155 expression levels increase as shown in Figure 1.

Consistent with these findings, exogenous expression of the TAp63 isoform caused miR-155 expression levels to decrease. Overexpression of the ΔNp63 isoform had no effect on miR-155 (Figure 2). These results indicate that TAp63 acts as a tumour suppressor and inhibits miR-155 expression.

Thus, it was unexpected that the ΔNp63 isoform, and not TAp63, bound directly to the p63 response element on the miR-155 host gene, as shown by ChIP assay of the miR-155 host gene p63 response element. On the basis of the results from both knockdown and overexpression of each isoform it would be predicted that TAp63 binds to the miR-155 host gene and suppresses its expression. However, the ChIP assays show ΔNp63 binding to the response element on *MIR155*, and that binding is enriched when TAp63 is knocked down (Figure 3). We speculate that when TAp63 is present, ΔNp63 is unable to bind to the miR-155 host gene. When TAp63 is removed, ΔNp63 can bind to the miR-155 host gene and drive expression as shown by luciferase assay (Figure 3). This is consistent with the observations in TAp63 knockdown cells that p63 binding to *MIR155* is enriched, and miR-155 levels are increased. This does seem to be conflicting with the finding that overexpression of ΔNp63 had no effect on miR-155 levels in BT549 cells (Figure 2), however miR-155 is already expressed at such a high level in this particular cell line [9] that any inductive effect of ΔNp63 is not displayed in this cell line.

Does the induction of miR-155 expression caused by the release from TAp63 regulation have any functional effect? To answer this question we performed scratch wound assays comparing the migratory potential of the TAp63 knockdown cells (indirectly overexpressing miR-155) with cells overexpressing miR-155 directly. Both TAp63 knockdown and miR-155 overexpression showed a significant increase in migration (Figure 4), indicating that miR-155, when released from TAp63 regulation, can drive migration. Further, in the presence of anti-miR-155 this phenotype was inhibited. This shows that it is the elevated expression of miR-155 that is the major driver of the phenotype resulting from TAp63 knockdown.

In a mouse xenograft model using the A431 cell line, both TAp63 knockdown and miR-155 overexpression enhanced tumour growth, while total p63 knockdown had no effect (Figure 5). Interestingly, TAp63 knockdown takes effect gradually on tumour growth and increases over time, whereas miR-155 overexpressing tumours have a significant (p<0.01) initial surge of growth early on with subsequent growth resulting in no significant difference from the scramble control. Despite this difference, both groups reach an average tumour volume of approximately 400 at day 21. This difference in growth dynamics could be due to a number of factors. It could be caused by variation between batches of animals used for the xenograft. Alternatively, it could be the impact of TAp63 knockdown on pathways other than those influenced by miR-155. Also of note is that the miR-155 levels in the miR-155 overexpression group are higher than in the TAp63 knockdown group even though the cell lines prior to the xenograft contained similar levels of miR-155. This could suggest a positive selection within the xenograft for cells overexpressing miR-155, consistent with miR-155's function as an antimiR. Alternatively, signals from the host animal might have interfered with the p63 interaction with the miR-155 host gene in some way that did not affect the direct miR-155 overexpressing cells. Regardless, the increased xenograft tumour growth in cells with TAp63 knockdown and miR-155 overexpression is consistent with miR-155 being a major contributor to the oncogenic phenotype driven by p63.

We therefore present a mechanism whereby TAp63 suppresses miR-155 expression and when this regulation is removed, ΔNp63 is able to drive miR-155 expression and thus migration and tumorigenesis. This mechanism is reflective of both the oncogenic properties of ΔNp63, and the tumour suppressor role of TAp63 [27, 29]. In addition, the ΔNp63 isoform is the most expressed isoform in many cancers [32], which may be linked to miR-155 being upregulated in many solid cancers [2-5]. The oncogenic effects of TAp63 knockdown are in line with the documented oncogenic role of miR-155 [6-8], and the attenuation of this effect by the anti-miR-155 antimiR indicate that the functional effect caused by TAp63 knockdown is mediated largely by miR-155. Further, the increased migration potential caused by TAp63 knockdown supports previous studies linking p63 to migration, where a microarray of p63-siRNA-treated squamous cell carcinoma showed the upregulation of multiple cell motility genes [33], and TAp63 regulates miR-205 which is linked to cell migration in prostate cancer [34].

As the two p63 isoforms are known to directly interact [35, 36], this provides a possible mechanism of the unexpected enrichment of ΔNp63 binding to the response element on the miR-155 host gene when TAp63 is knocked down. Although it is usually the ΔNp63 isoform that exerts a dominant negative effect on TAp63 [31], it is also possible that TAp63 could have a negative effect on ΔNp63 action. The final member of the p53 family is p73. It is possible that p73 is also involved in this pathway, as it has been shown that p63-regulated miRs other than miR-155 have been linked to p73, inhibiting its ability to promote genetic stability and chemosensitivity [37]. Exploring the relationship between miR-155 and p73 might be a valuable path for future research.

These results provide a potential mechanism for our previous findings that miR-155 expression is upregulated in the presence of mutant p53 [9], as mutant p53 interacts with and inhibits the action of p63 isoforms, in particular TAp63 [38-40]. Further, mutant p53 has been shown to use p63 as a molecular chaperone to promote the expression of target genes and induce invasion [41]. In this instance mutant p53 might be using the ΔNp63 isoform as a chaperone to drive a migratory phenotype. In the future the relationship between the p63 isoforms, mutant p53 and miR-155 should be investigated to reveal the full mechanism of miR-155 upregulation in cancer.

## MATERIALS AND METHODS

### Expression constructs

pMSCV-Puro-GFP, pMSCV-Puro-GFP-miR-155 – kindly donated by Erik Flemington TAp63-specific shRNA oligonucleotide (V2LHS24249), total p63 targeting shRNA (V2LHS24246 and V3LHS397885), non-silencing control (RHS4346) – Thermo Scientific pcDNA3.1-TAp63-HA, pcDNA3.1-ΔNp63-myc, pcDNA3.1 vehicle – kindly donated by Patricia Muller

### Cell culture

MCF10A cells were maintained in Dulbecco's modified Eagle's medium F12, supplemented with 5% horse serum, 20ng/mL epidermal growth factor (R&D Systems), 0.5μL/mL hydrocortisone (Sigma), 100ng/mL cholera toxin (Sigma) and 10μg/mL insulin (Promega). A431 and H1299 cells were maintained in DMEM supplemented with 10% foetal calf serum (FCS). BT549 cells were maintained in RPMI supplemented with 10% FBS.

For constitutive knockdown of p63 isoforms, TAp63-specific shRNA oligonucleotide (V2LHS24249) total p63 targeting shRNA (V2LHS24246 and V3LHS397885) or a non-silencing control (RHS4346) was used in accordance with the manufacturer's protocol (Open Biosystems).

For exogenous expression of p63 isoforms, 1 μg of pcDNA3.1 vehicle control, pcDNA3.1-TAp63-HA or pcDNA3.1-ΔNp63-myc was transfected using lipitoid transfection reagent following the manufacturer's protocol [42].

MCF10A and A431 cells were engineered to express either miR-155 or a non-targeting SCR control through retroviral mediated transduction with viruses generated by the pMSCV-Puro-GFP-miR-155 or pMSCV-Puro-GFP vectors according to manufacturer's instructions, followed by selection in 1μg/mL puromycin (Sigma-Aldrich, Castle Hill, NSW, Australia).

### Isolation of RNA, reverse-transcription (RT–PCR) and microRNA analysis

Briefly, total RNA was extracted from cells using Trizol (Invitrogen), with quantitative real-time PCR performed as previously described [9]. Specific primers for real-time PCR are listed in Supplementary Table 1. Mature miR-155 measurements were made using the ABI Taqman miRNA assay for miR-155 following the manufacturer's protocol. Mature miR-155 levels were normalized using the average expression levels of housekeeper miR-16.

### Western blot analysis and ChIP

Western blot analysis was performed as previously described [43], using rabbit α-p63 H-129 (Santa Cruz) and mouse α-β actin (Sigma Aldrich). ChIP analysis was performed as previously described [9].

### Luciferase assay

The wildtype or mutant miR-155 p63 response element was cloned into the pGL3 basic luciferase vector (Promega). Primers used for cloning the response element are listed in Supplementary Table 1. The luciferase vector was then transfected into 293T cells using lipitoid transfection reagent according to the manufacturer's protocol [42] along with 5 ng of pcDNA3.1 vehicle control, pcDNA3.1-TAp63-HA or pcDNA3.1-ΔNp63-myc and 20ng of pRL-TK. The assay was carried out using the Promega Dual Luciferase Reporter Assay System kit, using 20 uL of the LARII and Stop&Glo reagents and following the manufacturer's instructions. Ratio of firefly to renilla luciferase was measured using a Glomax 20/20 luminometer. Reactions were carried out in duplicate, and each well was measured twice to ensure correct reading.

### Migration assay

Cell migration was analysed by measuring the width of a scratch wound in real time using Incucyte (Essen, MI, USA). Phase contrast images were taken every 30 min or 1 hour and wound closure and cell confluence calculated using Incucyte software. For antimiR inhibition of miR-155, miRvana miR-155 inhibitor (Life Technologies) or a negative control was transfected using lipitoid transfection reagent following the manufacturer's protocol [42].

### Xenograft

Stably infected cells (5×10^5^ cells per animal) were suspended in 100uL of DMEM serum free medium, mixed with 100μL of 10% Matrigel (BD) and injected subcutaneously into 5-8 week old female BALB/c nude mice. Tumour size was measured twice weekly using callipers. Animals were humanely euthanized after 3-5 weeks depending on tumour volume. The tumour was snap frozen and RNA extracted for miR-155 measurement by specific Taqman probe. Animal protocols were approved by the ethics committee of University of Adelaide and IMVS.

## Supplementary Figures and Tables


