# Supplementary material for: TAp63 regulates oncogenic miR-155 to mediate migration and tumour growth
Source: Oncotarget. 2013 Aug 31;4(11):1894–903. doi: 10.18632/oncotarget.1228 (PMC3875757; doi:10.18632/oncotarget.1228)
Supplement: Supplementary file 1 [file oncotarget-04-1894-s001.docx]

Supplementary Data

Supplementary Figure 1

A: Relative p63 isoform expression in MCF10A and A431 cell lines as measured by realtime PCR using isoform specific primers. B: miR-155 expression in MDA-MB-231, BT549, MCF10A and A431 cell lines as compared to miR-16, measured by Taqman probe.

Supplementary Figure 2

Relative miR-155 expression in MCF10A TAp63 knockdown and miR-155 overexpressing cells, as measured by miR-155 specific Taqman probe, and compared to miR-16.

Supplementary Figure 3

A, B: The ability of MCF10A (A) or A431 (B) total p63 knockdown cells to migrate with application of anti-miR-155 inhibitor (+) or a non-targeting anti-miR (-) was determined by a scratch-wound assay using Incucyte (Essen). Wound width was calculated using Incucyte software.

Supplementary table 1 – Oligonucleotides

| Primer | Sequence |
| --- | --- |
| TAp63 realtime forward | GGACTGTATCCGCATGCAG |
| TAp63 realtime reverse | GAGCTGGGCTGTGCGTAG |
| DNp63 realtime forward | TTGTACCTGGAAAACAATGCCC |
| DNp63 realtime reverse | GGGACTGGTGGACGAGGAG |
| miR-155HG realtime forward | AGCAAGCGCGGGGAACCAAGG |
| miR-155HG realtime reverse | TCCATTGGGTGGGAGAGCCAAGG |
| miR-155 p63RE cloning forward HindIII | CCTGCTGGATaagcttAGACTTCAGG |
| miR-155 p63RE cloning reverse NcoI | TTATGAACAccatggTGAACAAGCCAAAACCTGCAatta |
| miR-155 p63RE cloning reverse mutant NcoI | TTATGAACAccatggTGaaTaaTccaaaaTctTcaatta |
